# Real-world data of autologous stem cell transplantation for patients with mantle cell lymphoma in Argentina

**DOI:** 10.1016/j.htct.2026.106449

**Published:** 2026-04-02

**Authors:** Martín Milanesio, Mariano Berro, Adriana Vitriu, María Sol Jarchum, Silvina Palmer, Amalia Cerutti, Evelyn Colombo, Malena Rocca, Lautaro Sardu, Ana Laura Romero, Micaela Quarchioni, Fernando Warley, Alejandra Banchieri, Germán Wernicke, María Marta Rivas, José Trucco, Leandro Castellanos, Luciana Guanchiale, Ana Romina Montivero, Cecilia Foncuberta, Ana Lisa Basquiera

**Affiliations:** a Hospital Privado Universitario de Córdoba, Córdoba, Argentina; b Instituto Universitario de Ciencias Biomédicas de Córdoba, Córdoba, Argentina; c Hospital Universitario Austral, Buenos Aires, Argentina; d Instituto Alexander Fleming, Buenos Aires, Argentina; e Sanatorio Allende, Córdoba, Argentina; f Hospital Británico, Buenos Aires, Argentina; g Sanatorio Británico, Rosario, Argentina; h Hospital el Cruce, Buenos Aires, Argentina; i Hospital Italiano de Buenos Aires, Buenos Aires, Argentina; j Hospital de Clínicas, Buenos Aires, Argentina

**Keywords:** Mantle cell lymphoma, Bone marrow transplant, Consolidation chemotherapy

## Abstract

**Background:**

For eligible patients with mantle cell lymphoma who respond to induction therapy, first-line consolidation with autologous hematopoietic progenitor cell transplantation remains a standard treatment. However, outcomes for these patients in Argentina have not been fully characterized. This paper aims to describe the factors linked to improved survival of patients with mantle cell lymphoma after transplantation in Argentina. In addition, the association between relapse within the first 24 months after transplant and overall survival was evaluated.

**Methods:**

A retrospective, multicenter study was carried out. Patients over 18 years of age with a diagnosis of mantle cell lymphoma who received autologous hematopoietic progenitor cell transplantation from 2007–2023 at centers affiliated with the Argentine Group for Bone Marrow Transplantation and Cellular Therapy (GATMO-TC) were included. For the survival analysis, a landmark approach was utilized: overall survival was calculated from the date of progression for the group that relapsed within 24 months, and from the 24-month post-transplantation landmark for those who did not.

**Results:**

One hundred and sixty-six patients from nine Argentine centers were included, 128 of whom were men (77%). The median age at transplantation was 58 years. Eighteen (11%) had blastoid morphology. The pretransplant status was complete response in 145 (87%) patients. With a median follow-up of 38.4 months, the median overall survival and progression-free survival were 102 and 48.8 months, respectively. In the multivariate analysis, the blastoid variant, an age ≥55 years, and a transplant comorbidity index ≥2 were independent predictors of survival.

**Conclusions:**

>70% achieved prolonged survival. Blastoid morphology, age older than 55 years, and comorbidities diminished outcomes after transplantation.

## Introduction

The current treatment of mantle cell lymphoma (MCL) for clinically suitable patients consists of a sequence that includes induction chemotherapy based on rituximab and high-dose cytarabine (HiDAC), followed by consolidation with autologous stem cell transplantation (ASCT) in those patients who achieve response with induction, and subsequently maintenance with rituximab. This sequence is associated with overall responses in >90% of patients, with complete response (CR) rates of 70% and four-year progression-free survival (PFS) of 83%. However, relapses may still occur after five years of remission [[1], [2], [3], [4], [5]].

The indication for ASCT as first-line consolidation is based on the trial by Dreyling et al., which compared consolidation with ASCT versus interferon alpha in patients with MCL after induction with a regimen similar to cyclophosphamide, doxorubicin, vincristine, and prednisone (CHOP) [6]. This study demonstrated a longer PFS for patients who underwent ASCT after their first remission, and subsequently also demonstrated a longer overall survival (OS) in favor of ASCT. In addition, ASCT was associated with a low mortality rate (5%), and patients who consolidated in CR had a better PFS than those who did so in partial response (PR) [7].

Despite the clear benefit of consolidating with ASCT in first remission when using CHOP-like induction regimens, this benefit was not demonstrated in randomized clinical trials after induction with modern regimens based on rituximab and HiDAC, which are currently used as first-line treatment for MCL [7]. Moreover, a subsequent analysis of the Dreyling et al. study found no differences in PFS between the two consolidation arms (ASCT versus interferon alpha) among patients who had received rituximab in induction [7]. Recently published results of the TRIANGLE clinical trial demonstrated no inferiority in the omission of ASCT when ibrutinib is added to first-line chemoimmunotherapy [8]. These are the main reasons why the percentage of patients with MCL receiving ASCT is currently decreasing [9]. However, there are Phase II and III trials that have demonstrated the effectiveness and safety of ASCT after induction with these modern regimens (rituximab and HiDAC), with very prolonged remissions, which could imply a cure in a considerable percentage of patients [2,4,5,[10], [11], [12]].

Given the uncertainty surrounding the outcomes of ASCT in patients with MCL and the lack of data on this topic in Argentina, a multicenter retrospective analysis was conducted. Patients diagnosed with MCL who underwent ASCT, both as first-line treatment and in the setting of relapse, at transplant centers affiliated with the Argentine Group for Bone Marrow Transplantation and Cell Therapy (GATMO-TC), were included. The objective of the study was to evaluate OS and PFS after ASCT, the cumulative incidence of relapse, and non-relapse mortality.

### Patients and methods

An analytical, retrospective, multicenter study was conducted, including all patients with MCL who received ASCT in nine transplant centers affiliated with GATMO-TC. The different variables were collected from each patient's medical record by a referring physician at each hospital. Subsequently, the data were centralized and analyzed in a tertiary care hospital in Córdoba, Argentina (Hospital Privado Universitario de Córdoba). The inclusion criteria were: i) diagnosis of MCL according to WHO 2017 criteria [13], ii) age equal to or >18 years at the time of diagnosis of the disease, iii) having received ASCT, either as first-line or subsequent line therapy, between January 1, 2007, and April 30, 2023, at transplant centers affiliated with GATMO-TC. The exclusion criteria were: i) not having achieved at least a partial response prior to transplant; ii) having received an allogeneic bone marrow transplant prior to autologous transplant; iii) age equal to or >76 years.

For patients who received induction therapy at an external center, the referring physician provided a comprehensive clinical history. This summary included the diagnostic methods used at disease onset, the various treatments administered, and the subsequent response to therapy; this information was then integrated into the patient’s medical record at the ASCT center. The response achieved before transplantation was assessed by physical examination and imaging, and was defined as CR if symptoms and tumor masses due to the disease disappeared, or as PR if tumor masses decreased by >50% with no new lesions. The types of conditioning used were defined by each center. Post-transplant follow-up was conducted according to institutional protocols. The frequency of surveillance imaging and the administration of rituximab maintenance therapy were left to the discretion of the treating physicians. Relapse and progression were defined as the appearance of new biopsy-confirmed disease or a 50% increase in the size of previously known lesions in patients who had achieved CR or PR [14]. Patients who continued follow-up outside the transplant hospital were contacted by telephone to assess their current status and their post-transplant therapies, including rituximab maintenance.

This study was carried out in full accordance with the current national and international regulations: Declaration of Helsinki of the World Medical Association in its most updated version, resolution 1480/2011 of the National Ministry of Health and the Law 25,326 on Protection of Personal Data. All the study data was treated with the utmost confidentiality and anonymously, with access restricted only to authorized personnel for the purposes of the study and, once more, in accordance with the aforementioned regulations. The study was approved by the Health Research Ethics Committee of the Hospital Privado Universitario de Córdoba.

### *Statistical analysis*

Continuous variables were expressed as median and range or mean and standard deviation according to distribution, and comparisons were performed using the Student’s t-test or the Mann-Whitney test, depending on their homogeneity. Categorical variables were expressed as number and percentage and analyzed using the chi-square test or Fisher's exact test according to expected frequencies. PFS was calculated from the date of transplant to the date of relapse, progression, or death, whichever occurred first. OS was calculated from the date of transplant to the date of death or last follow-up. Survival curves were estimated using the Kaplan-Meier method and compared across strata using the log-rank test. The cumulative incidence of relapse and non-relapse mortality were analyzed by competing risks. Patients who relapsed within 24 months of ASCT were categorized as POD24-positive. For the survival analysis, a landmark approach was used: overall survival for the POD24 group was calculated from the date of relapse or progression, while for the non-POD24 group, survival was calculated starting from the 24-month post-transplant landmark. Patients who died without progression and those who did not reach 24 months of follow-up were excluded. A probability value <0.05 was considered significant. Statistical analysis was performed using the EZR statistical program (Easy R).

## Results

From January 1, 2007, to April 30, 2023, 166 patients diagnosed with MCL underwent ASCT at nine Argentine centers belonging to the GATMO-TC group. The patients' baseline characteristics are shown in Table 1.

**Table 1:** Baseline characteristics of 166 patients who received Autologous stem cell transplantation for mantle cell lymphoma.

Characteristic	n (%)
**Male**	128 (77)
**Female**	38 (23)
**Age at diagnosis - median (range) years**	56 (37–69)
**Stage at diagnosis**	
I	4 (2)
II	6 (4)
III	22 (14)
IV	122 (73)
Unknown	12 (7)
**BM compromise at diagnosis**	107 (64)
**B symptoms at diagnosis**	62 (37)
**MIPI**	
Low	31 (18)
Intermediate	36 (22)
High	28 (17)
Unknown	71 (43)
**MIPIb**	
Low	9 (5)
Intermediate	16 (10)
High	27 (16)
Unknown	114 (69)
**Initial treatment at transplant center**	67 (40)
**Pre-transplant induction schedules**	
R-CHOP/R-DHAP	94 (57)
R-CHOP	32 (19)
R-hyper-CVAD	20 (12)
Others	20 (12)
**Rituximab pre-transplant**	165 (99)
High-dose cytarabine pre-transplant*	140 (84)

⁎ Includes patients who received high-dose cytarabine prior to R-CHOP.

### *Characteristics at the time of mantle cell lymphoma diagnosis*

Seventy-seven percent (*n* = 128) were men. The median age at diagnosis of MCL was 56 years (range: 37–69 years). Of the total, 144 (87%), 107 (64%), 62 (37%), and 18 (11%) had Stage III–IV disease, bone marrow involvement, B symptoms, and blastoid morphology, respectively. Prior to transplant, 165/166 (99%) and 140/166 (84%) received rituximab and HiDAC, respectively. Other characteristics are summarized in Table 1.

ASCT: autologous hematopoietic progenitor cell transplantation; MCL: Mantle Cell Lymphoma; BM: bone marrow; MIPI: Mantle Cell Lymphoma International Prognostic Index; MIPIb: biological Mantle Cell Lymphoma International Prognostic Index; R-CHOP: rituximab, cyclophosphamide, doxorubicin, vincristine, and prednisolone; R-DHAP: rituximab, cisplatin, cytarabine, and dexamethasone.

### *Characteristics at the time of transplant*

The characteristics of the patients at the time of ASCT are summarized in Table 2.

**Table 2:** Characteristics at the time of transplant.

Characteristic	
**Age at transplant - median (range) years**	58 (37–69)
**Transplant comorbidity index - median (range)**	0 (0–9)
**Pre-transplant lines - median (range)**	1 (1–5)
**Pre-transplant disease status**	
Complete response - n (%)	145 (87)
Partial response - n (%)	17 (10)
Unknown - n (%)	4 (2)
**Transplant in first remission - n (%)**	139 (83)
**Transplant in second remission - n (%)**	27 (16)
**Conditioning**	
BEAM - n (%)	98 (59)
CBV - n (%)	31 (19)

BEAM: carmustine, etoposide, cytarabine, and melphalan; CBV: cyclophosphamide, carmustine, and etoposide.

### *Post-transplant results*

Following ASCT, 58 (35%) patients received rituximab maintenance therapy. Disease status at Day +100 post-transplant was CR (*n* = 146; 88%), PR (*n* = 2; 1%), progressive disease (*n* = 4; 2%), and unknown (*n* = 14; 9%).

### *Overall and progression-free survival post-transplant*

**Fig. 1:**
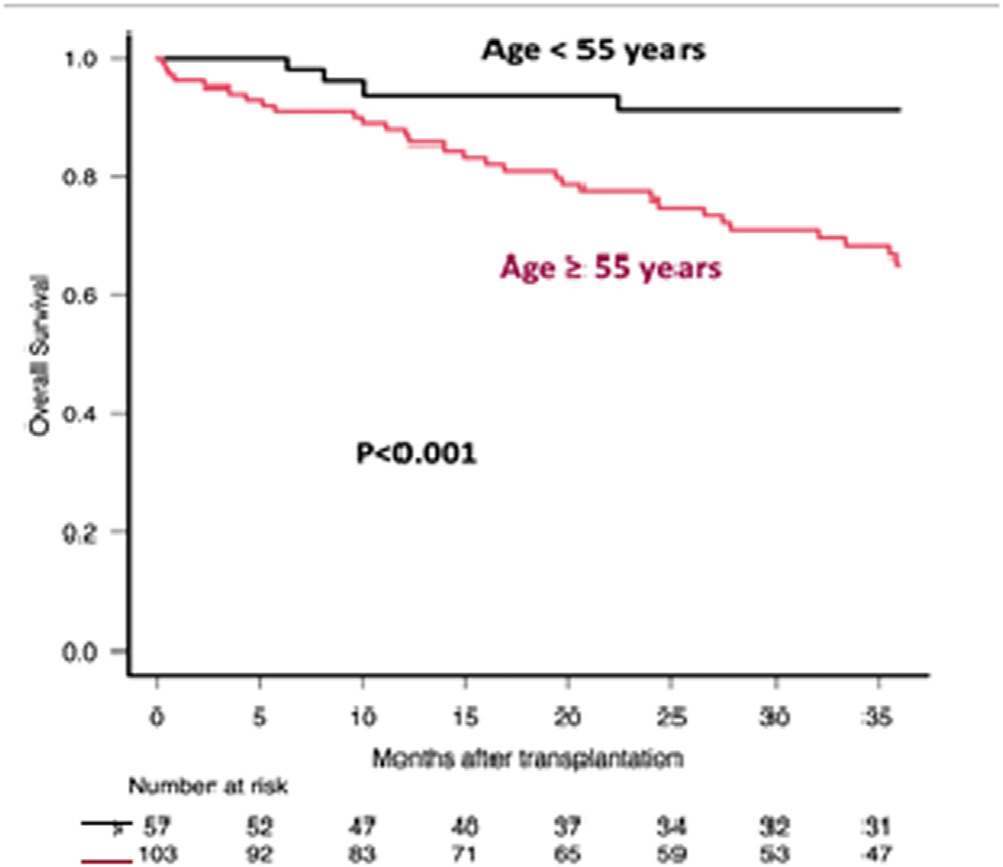
Overall survival (OS) after Autologous Hematopoietic Progenitor Cell Transplantation (ASCT) according to age at transplantation. Comparison between patients aged ≥55 years and those aged <55 years.

**Fig. 2:**
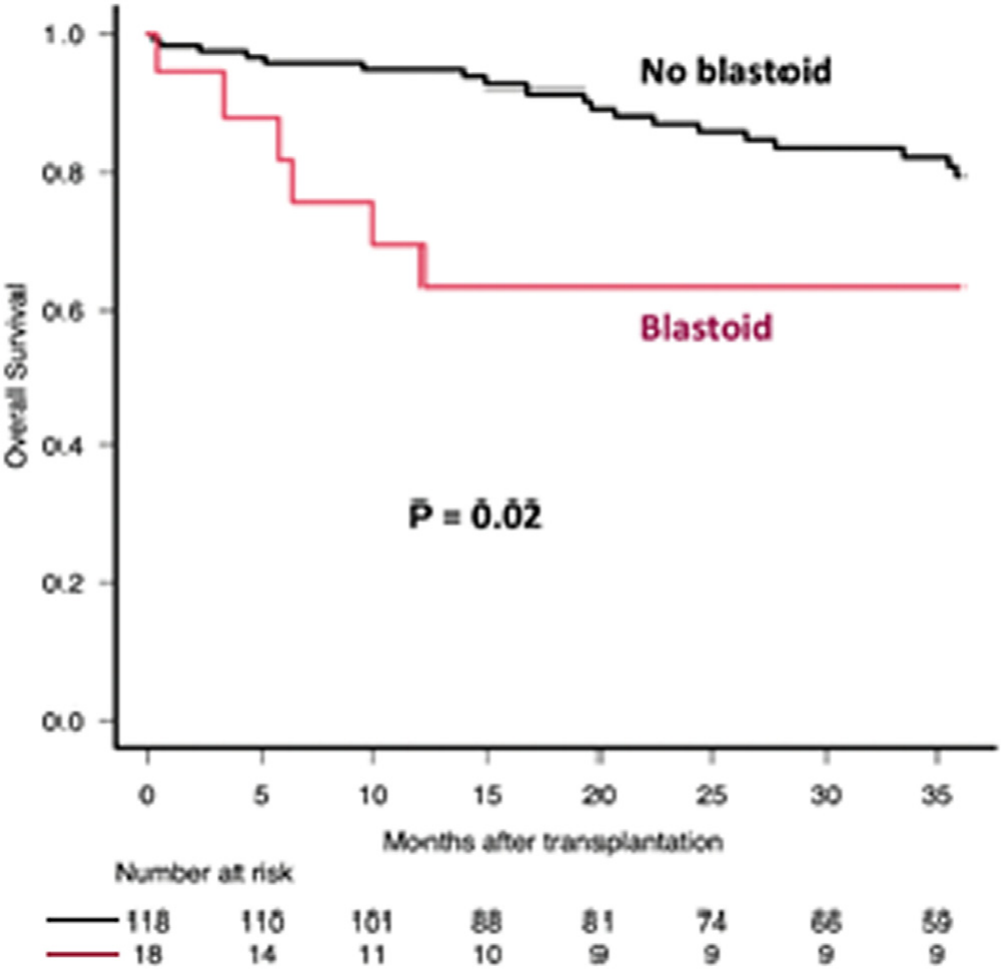
Overall survival (OS) after Autologous Hematopoietic Progenitor Cell Transplantation (ASCT) according to morphology. Comparison between patients with and without blastoid morphology.

**Fig. 3:**
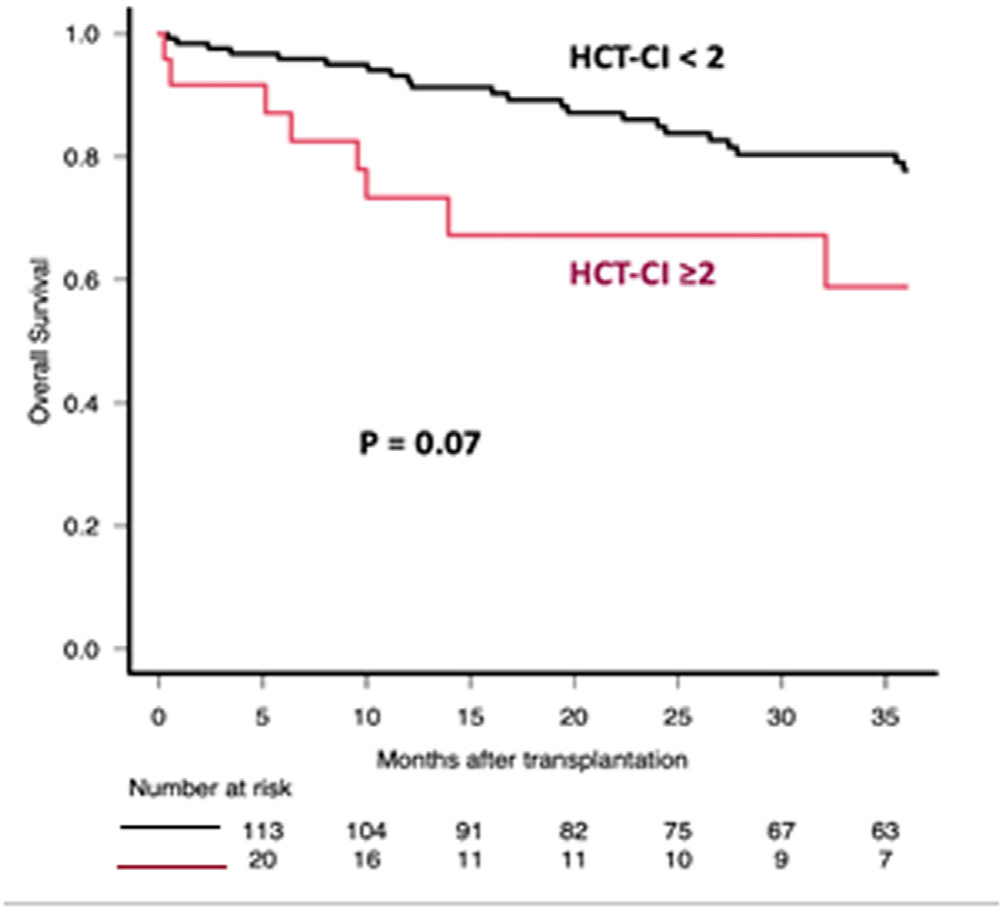
Overall survival (OS) after Autologous Hematopoietic Progenitor Cell Transplantation (ASCT) according to HCT-CI at transplantation. Comparison between patients with an Hematopoietic Cell Transplantation Comorbidity Index (HCT-CI) ≥2 and those with an HCT-CI <2.

After a median follow-up of 38.4 months (interquartile range: 14.2–71.2), the median OS and PFS were 102 (range: 85.5-not reached months) and 48.8 months (range: 41.2–74 months), respectively. The estimated 3-year OS and PFS were 74.9% (95% confidence interval: 66.5–81.5) and 62.1% (95% confidence interval: 53.1–69.8), respectively. In the multivariate analysis, the blastoid variant (Hazard ratio [HR]: 5.81; p-value <0.001), age ≥55 years (HR: 3.95; p-value = 0.006) and transplant comorbidity index ≥2 (HR: 3.09; p-value = 0.008) were independent predictors of OS (Fig. 1, Fig. 2, Fig. 3). The 3-year OS for those transplanted in CR was 76% versus 55% for those transplanted in PR (p-value = 0.064). While OS was similar for those who received rituximab maintenance after ASCT versus those who did not, the 3-year PFS for those who received rituximab maintenance was 66% versus 49% for those who did not (p-value = 0.016). Induction and conditioning regimens, pre-transplant response (CR versus PR), B symptoms, elevated mantle cell lymphoma international prognostic index, and Stage III-IV did not affect OS or PFS.

### *Post-transplant relapse*

**Fig. 4:**
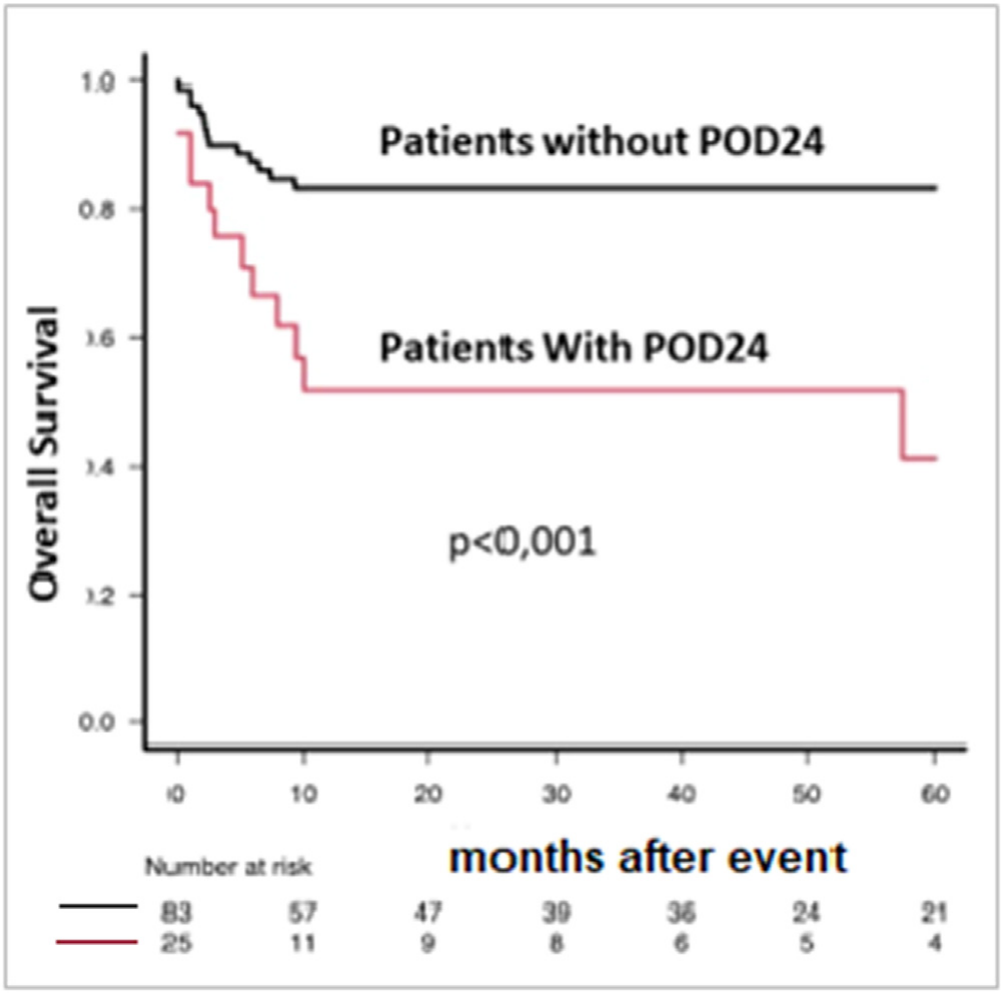
Overall survival (OS) according to progression of disease within 24 months (POD24) status. Comparison between patients with and without relapse within 24 months after autologous hematopoietic progenitor cell transplantation (ASCT). For this landmark analysis, OS was calculated from the date of relapse or progression for the POD24 group and from the 24-month post-ASCT landmark for the group without POD24.

Of the total number of patients, 56 (34%) relapsed. The cumulative incidences of relapse at 12, 24, and 36 months were 9.1% (5.2–14.4%), 17.4% (11.7–24.1%), and 25.3% (18.2–33%), respectively. In the multivariate analysis, the only factor independently associated with a higher cumulative incidence of relapse was having undergone ≥2 lines of therapy prior to ASCT (p-value ≤0.001). Within the first 24 months post-ASCT, 25 patients relapsed (POD24), of whom 14 died. The 1-year and 5-year overall survival rates were significantly lower for the POD24 group (51.6% and 41.3%) compared to those without a POD24 event (83.3% and 83.3%, respectively (p-value <0.001; Figure 4).

### *Post-transplant mortality*

Of the total, 51 (31%) patients died, with 24/51 in remission. The cumulative incidence of non-relapse mortality at 3, 6, and 12 months was 3% (range: 1.1–6.5%), 4.9% (range: 2.3–9.9%), and 6.3% (range: 3.2–10.8), respectively. In multivariate analysis, blastoid morphology (HR: 3.28; p-value = 0.014) and age ≥55 years (HR: 2.63; p-value = 0.013) were predictors of non-relapse mortality. The causes of non-relapse mortality were: sepsis (*n* = 7), COVID-19 (*n* = 6), infectious pneumonia (*n* = 2), acute pulmonary thromboembolism (*n* = 1), infectious meningoencephalitis (*n* = 1), candidiasis (*n* = 1), glioblastoma (*n* = 1), and unknown (*n* = 5). One case of acute myeloid leukemia and one case of myelodysplastic syndrome following ASCT were reported. More than five years after ASCT. Twenty-three patients are alive and relapse-free.

## Discussion

The outcomes with MCL after ASCT in Argentina are presented in this retrospective study.

The baseline characteristics of the cohort were similar to those reported in different studies of patients with MCL who were candidates for ASCT, with a median age at diagnosis of approximately 56–58 years, a high predominance of males, and Stage III-IV disease with bone marrow involvement. In line with other studies, B symptoms were present in 37%, blastoid morphology in 11%, and high mantle cell lymphoma international prognostic index in 17% of cases [2,4]. Unlike other studies, the induction regimens used did not impact post-ASCT OS. This may be because the majority of the patients received HiDAC prior to ASCT (84%). Moreover, unlike the study by Delarue et al. [5], patients who presented with B symptoms at diagnosis did not have worse OS in the present study.

With a median follow-up of 38 months, the 3-year PFS and OS were 62% and 75%, results similar to those observed by Touzeau et al. [15], but somewhat lower than those of Merryman et al. [4], who reported 3-year PFS and OS of 83% and 92%, respectively after induction with rituximab-bendamustine alternating with rituximab-cytarabine and subsequent consolidation with ASCT. Similarly, the current OS results were lower than those of Hermine et al. [11] after a long-term follow-up of patients who underwent R-CHOP/R-DHAP followed by ASCT. These authors reported a five-year OS of 76%. We believe the differences in OS observed in the present study are associated with a higher transplant-related mortality, which may be explained by the inclusion of patients over an extended period of time. The lower transplant-related mortality observed in trials is likely attributable to strict inclusion criteria, which may not accurately reflect the complexities of a real-world patient population. In this regard, in a real-world report of patients with MCL who underwent ASCT, survival rates were similar to those found in the present series [16]. Although the results of the TRIANGLE clinical trial [8] demonstrated that adding ibrutinib to induction chemotherapy, followed by ASCT and then maintenance employing ibrutinib for two years, provides survival benefits, it has not yet been demonstrated that, with the addition of ibrutinib, ASCT can be omitted. The use of ibrutinib in first-line treatment was associated with increased hematologic and infectious adverse events [8].

As in most studies that include rituximab and HiDAC in induction regimens, most of the current patients were transplanted in CR which was associated with longer OS. As in other studies [15], the blastoid variant and age were clear predictors of OS.

For indolent lymphoma, such as follicular lymphoma, progression or death within 24 months of treatment initiation, is an indicator of poor survival [17]. In MCL, retrospective studies identified POD24 as associated with short OS [18,19]. However, the predictive value of POD24 for OS still needed to be assessed, using data from patients included in clinical trials. Recently, an analysis of previously untreated MCL patients enrolled in six multicenter trials conducted in France demonstrated that POD24 status is strongly associated with subsequent OS in MCL and that rituximab maintenance provided significant protection against the risk of POD24 [20]. In the present study, the OS of patients with POD24 was much lower than those who did not experience the event.

Regarding relapse, the cumulative incidences of relapse of this study were 9% and 25% at 12 and 36 months, respectively, both values lower than those reported in other studies [15]. The non-relapse mortality in the present series was 6% at one year and the main causes of non-relapse mortality were infections, as was reported in a Spanish study [21].

A positive feature of this study is the large number of patients analyzed. However, primarily due to its retrospective design, this study has several limitations, most notably missing data for variables such as Ki-67. Consequently, the biological Mantle Cell Lymphoma International Prognostic Index could not be calculated for the majority of the study population.

## Conclusion

These results of ASCT for MCL in Argentina are encouraging, with >70% of patients achieving prolonged survival. Therefore, we believe this therapy still has a key role as a first-response consolidation therapy in the country. Certain factors, such as the number of prior lines ≥2, age over 55 years, and comorbidities, diminish the results after ASCT. The benefit of consolidation with ASCT is less in patients with blastoid morphology, thus another more aggressive consolidation strategy should be sought for these patients. Early post-transplant relapse (POD24) confers a poor prognosis with these patients urgently requiring aggressive strategies.

## Funding sources

This research did not receive any specific grant from funding agencies in the public, commercial, or not-for-profit sectors.

## Data availability

The data that support the findings of this study are available from the corresponding author upon reasonable request.

## Conflicts of interest

None to declare.
